# Comprehensive analysis of brain injury parameters in a preclinical porcine model of acute liver failure

**DOI:** 10.3389/fmed.2024.1363979

**Published:** 2024-03-28

**Authors:** Philipp Felgendreff, Seyed M. Hosseiniasl, Lisa Felgendreff, Bruce P. Amiot, Anna Minshew, Boyukkhanim Ahmadzada, Zhi Qu, Silvana Wilken, Ines Arribas Gomez, Scott L. Nyberg, Casey N. Cook

**Affiliations:** ^1^Department of Surgery, Mayo Clinic, Rochester, MN, United States; ^2^Department of General, Visceral, and Transplantation Surgery, Hannover Medical School, Hannover, Germany; ^3^Department of Journalism and Communication Research, Hannover University of Music, Drama, and Media, Hanover, Germany; ^4^Transplant Center, Hannover Medical School, Hannover, Germany; ^5^Department of Neuroscience, Mayo Clinic, Jacksonville, FL, United States; ^6^William J. von Liebig Center for Transplantation and Clinical Regeneration, Mayo Clinic, Rochester, MN, United States

**Keywords:** brain injury, acute liver failure, serum parameter, porcine model, GFAP

## Abstract

**Introduction:**

Acute liver failure (ALF) is defined as acute loss of liver function leading to hepatic encephalopathy associated with a high risk of patient death. Brain injury markers in serum and tissue can help detect and monitor ALF-associated brain injury. This study compares different brain injury parameters in plasma and tissue along with the progression of ALF.

**Method:**

ALF was induced by performing an 85% liver resection. Following the resection, animals were recovered and monitored for up to 48 h or until reaching the predefined endpoint of receiving standard medical therapy (SMT). Blood and serum samples were taken at T_baseline_, T_24_, and upon reaching the endpoint (T_end_). Control animals were euthanized by exsanguination following plasma sampling. Postmortem brain tissue samples were collected from the frontal cortex (FCTx) and cerebellum (Cb) of all animals. Glial fibrillary acidic protein (GFAP) and tau protein and mRNA levels were quantified using ELISA and qRT-PCR in all plasma and brain samples. Plasma neurofilament light (NFL) was also measured using ELISA.

**Results:**

All ALF animals (*n* = 4) were euthanized upon showing signs of brain herniation. Evaluation of brain injury biomarkers revealed that GFAP was elevated in ALF animals at T_24h_ and T_end_, while Tau and NFL concentrations were unchanged. Moreover, plasma glial fibrillary acidic protein (GFAP) levels were negatively correlated with total protein and positively correlated with both aspartate transaminase (AST) and alkaline phosphatase (AP). Additionally, lower GFAP and tau RNA expressions were observed in the FCTx of the ALF group but not in the CB tissue.

**Conclusion:**

The current large animal study has identified a strong correlation between GFAP concentration in the blood and markers of ALF. Additionally, the protein and gene expression analyses in the FCTx revealed that this area appears to be susceptible, while the CB is protected from the detrimental impacts of ALF-associated brain swelling. These results warrant further studies to investigate the mechanisms behind this process.

## Introduction

Acute liver failure (ALF) results from sudden and severe hepatocellular injury in patients without preexisting liver diseases. The most common etiology of ALF includes drug-induced liver injury, hepatitis virus infection, and post-operative, and autoimmune liver failure (1, 2).

Regardless of the etiology, ALF causes significant impairment of the anabolic, catabolic, and detoxification functions of the liver. This leads to a rapid progression of the disease with an overall mortality rate of 30–75% (3). The only available treatment options to save these patients are orthotopic liver transplantation and intensive care monitoring combined with standard medical treatment (SMT) to stabilize liver function. Due to the general donor organ shortage, liver transplantation procedures represent a treatment option for only a limited number of patients (4). For the remaining patients receiving SMT, timely detection and treatment of ALF-associated complications are essential for improving the survival rate.

Next to coagulopathy, systemic inflammation, renal and pulmonary dysfunction, and impaired detoxification function of the injured hepatocytes represent the leading complications in ALF patients (5). The reduced detoxification function of the liver leads to hyperammonemia, which is the leading cause of death in ALF patients (6–8). Hyperammonemia impairs the blood–brain barrier with subsequent astrocyte edema and brain swelling, leading to an increase in intracranial pressure (ICP). As this cascade advances, ischemic brain injury and brain herniation occur, ultimately leading to the inevitable death of the patient (1). Therefore, the detection of brain injury caused by ALF and the prevention of cerebral edema are crucial aspects in managing ALF patients (9).

However, the objective assessment of hepatic encephalopathy in patients with ALF is challenging in clinical practice. Most patients need extensive cardiopulmonary support requiring sedation and mechanical ventilation. Furthermore, clinical signs of brain herniation become apparent only in the late stage of the disease, posing a challenge for the early detection of ALF-related brain injury (10). Therefore, multiple direct and indirect ICP measuring devices were established to monitor the ICP accurately in patients with ALF.

The most standardized method of measuring the brain parenchymal pressure is by drilling a hole in the skull and placing an ICP probe directly in the parenchyma or the ventricles (11). However, this invasive method of measuring ICP directly can be associated with multiple adverse events, such as probe-associated infections or hemorrhages (12). Given the coagulopathy associated with ALF, the risk of ICP-caused hemorrhage can significantly increase from 5.7 to 10.2% in ALF patients (13, 14). To minimize complications in this patient group, indirect ICP measurement methods, such as determining the optic nerve sheath diameter (ONSD), have been used to screen for elevated ICP (15).

However, the prognostic value of these indirect ICP measuring devices is currently uncertain (16, 17). In contrast, non-invasive serum biomarkers such as glial fibrillary acidic protein (GFAP), neurofilament light (NFL) chain protein, tau, and S100B have shown value as diagnostic indicators (18). The most commonly used serum biomarker in humans is S100B, which has been shown to correlate with acute brain damage, including traumatic and ischemic brain injury (19, 20) as well as ALF in patients (20). Additionally, serum GFAP levels are widely accepted as a biomarker of clinical severity and the extent of intracranial pathology after traumatic brain injury. Furthermore, there is growing evidence supporting the potential clinical use of serum GFAP levels in neuroinflammatory and neurodegenerative diseases (10). Of particular relevance to the current study, a recent report found that serum GFAP was increased in patients with hepatic encephalopathy associated with cirrhosis (21). However, it remains unclear whether concentrations of tau or NFL in the blood might be altered in patients with ALF-induced brain injury. Notably, of potential biomarkers, only the serum S100B levels in ALF-associated brain injury have been investigated in a non-human primate model (22). Therefore, this study aimed to analyze the protein concentrations of tau, NFL, and GFAP in the blood of an established porcine ALF model to assess the early diagnostic value of these parameters in the context of ALF-associated brain injury and to investigate molecular changes in the brain to provide insight into the neurological consequences of ALF.

## Method

### Study design

Teen domestic wild pigs (30–60 kg) were obtained from a local vendor (Manthei Hog Farm, Elk River, MN) and randomized in two study groups (ALF group (*n* = 4) and control group (*n* = 6)). The design of the study and the involved animal study were approved by the Institutional Animal Care and Use Committee (No: A6726-22) of the Mayo Clinic.

In all ALF pigs, an ambulatory ICP probe and double-lumen venous catheter were placed under general anesthesia 3 to 4 days prior to 85% liver resection surgery. On the day of ALF induction, an 85% liver resection was performed, followed by the following sequence of events: blood draw and ICP measurement (T = baseline), pre-hepatectomy CT scan (T = PreCT), 85% hepatectomy, and posthepatectomy CT scan (T = post-CT). The completion of 85% liver resection was defined as T0 = 0 h. Following the 85% liver resection, all ALF animals were recovered for the first 24 h in the post-anesthesia recovery room and underwent SMT. Twenty-four hours after surgery (T_24h_), the animals were again sedated and transferred to the operation room for invasive hemodynamic monitoring, transvaginal urine catheter placement, extended intravenous fluid application in combination with continued SMT for up to 48 h after ALF induction, or until reaching predefined endpoints of the study (T_end_). In the six control group animals, only a laparotomy with blood draw (T = baseline) and subsequent euthanasia were performed.

### Anesthesia and surgical procedures

The surgical procedures were performed under general anesthesia induced with an intramuscular injection of Telazol (5 mg/kg), Xylazine (2 mg/kg), and Buprenorphine (0.3 mg/kg). Following intubation, the general anesthesia was maintained with 1–3% inhaled isoflurane. During therapy, Buprenorphine (up to 0.3 mg/kg) was administered to each animal in the group with an interval of 8-12 hours.

### Placement of intracranial pressure probe and double-lumen central venous catheter

The ICP probe and the double-lumen venous catheter placement were performed in a single session, as previously described (23). All pigs were placed in the prone position, and a 3 cm-diameter semi-circular skin incision was made on the skull just above the level of the eyes. After exposing the skull, a 5 French (~4 mm) burr hole was drilled through the skull. The tip of the implantable ICP probe was placed directly in the brain tissue, and its transmitter was positioned in the subcutaneous space. Following this step, a cuffed central venous catheter (CVC) was inserted into the right jugular vein under ultrasound guidance by using the Seldinger technique. The position of the catheter tip in the right atrium was confirmed by fluoroscopy. The catheter was tunneled subcutaneously to the pig’s back and exited at the level of the scapula.

### 85% hepatectomy

All ALF animals underwent 85% hepatectomy, according to the description by Chen et al. (23). For this procedure, the sedated pigs were placed in the spine position on the operation table. Following the laparotomy, the left and right lateral liver lobes, the PV, hepatic artery, and hepatic veins were identified and isolated. To minimize blood loss during resection, the left and middle lobe PVs were isolated and ligated with 2-0 silk. Hepatectomy was performed from left to right, removing the left lateral lobe, both left and right medial lobes, and the majority of the right lateral lobe; the caudate lobe and a small part of the right lateral lobe were left as 15% remnant liver parenchyma. The clamp and crush technique was used to complete the parenchymal transection. The abdominal wall fascia, abdominal wall, and skin were closed in layers, and the extent of hepatectomy was confirmed by CT volumetry comparing the native (pre-resection) liver volume to the remnant (post-resection) liver volume.

### Standard medical therapy (SMT) after 85% hepatectomy

All ALF animals were transferred to the post-anesthesia recovery room for the first 24 h. Continuous IV 5% dextrose normal saline (D5NS) was applied during this period to maintain a physiological blood glucose level. Additionally, every 2 h, the blood glucose level and the ICP were measured. In cases where the blood glucose level was less than 75 mg/dL, 5 mL of 50% dextrose was applied.

Twenty-four hours after the 85% hepatectomy was completed, all animals underwent the following sequence of events prior to the transfer on the operation table: induction of sedation by using propofol infusion at 80 mcg/Kg/min, intubation, urine catheter placement, Blood pressure, heart rate, oxygen saturation and ICP were monitored continuously. Intravenous fluid, including D5NS and PlasmaLyte, was given together at a minimum rate of 100 mL/h to a maximum rate of 400 mL/h to maintain the mean arterial pressure (MAP) above 50 mmHg and blood glucose above 75 mg/dL. Dobutamine (2–10 mcg/kg/min) and phenylephrine (0.5–3 mg/kg/min) were administered when the MAP dropped below 50 mmHg despite receiving the maximum amount of IV fluid. Mechanical ventilation was needed if the end-tidal PCO_2_ was more than 50 mmHg or the oxygen saturation was less than 92% despite the use of oxygen.

### Study endpoints

The following humane endpoints were used to terminate the investigation:

- Survival to 48 h after hepatectomy
- Signs of hepatic encephalopathy, stage III or IV
- Elevated ICP (>20 mmHg) for over 1 h
- Mean arterial blood pressure < 30 mmHg x 60 min at maximum vasopressor support (PlasmaLyte flow rate: 400 mL/h, dobutamine 10 mcg/kg/min, and phenylephrine 3 mg/kg/min).

### Blood sampling in the ALF pigs

Blood samples were taken from the central line prior to the 85% hepatectomy (T_baseline_), at T_24h_ and the T_end_ of the ALF. In these samples, clinical chemistry parameters (aspartate transaminase (AST), alanine transaminase (ALT), alkaline phosphatase (AP), international normalized ratio (INR), total protein (TP), total bilirubin, creatinine, and ammonia concentration) were measured using the Piccolo Xpress (Abaxis, Union City, CA). The central blood count (CBC) was detected using the Abaxis HM5 VetScan Hematology Analyzer (Axonia Medial, Singapore).

Additional plasma samples were collected from the ALF pigs at T_baseline_, T_24h_, and the T_end_ to conduct the ELISA analysis of brain plasma parameters.

### Necropsy and brain sampling

Following the blood sampling procedure, or after the ALF animals had reached their endpoint and were euthanized, all animals were moved to the necropsy room for brain tissue sampling. The skin above the eyes was removed to expose the entire skull. Using an oscillating bone saw, the scull in this area was inserted in a hexagon shape, exposing the frontal lobe protected by the dura. Following the insertion and removal of the dura, fresh frozen tissue samples were taken from the frontal cortex (FCTx) of each animal. To obtain fresh frozen tissue samples from the cerebellum (Cb), the cerebrum was removed and the tentorium cerebelli dissected to enable tissue sampling from the Cb.

### Preparation of brain homogenates

Approximately 60–80 mg frozen brain tissue was homogenized in a 10x volume of Co-IP buffer (50 mM Tris, 274 mM NaCl, 5 mM KCl, 5 mM EDTA, 1% Triton X-100) with 1 mM PMSF and protease and phosphatase inhibitor cocktails. Following sonication, samples were centrifuged at 20,000 g for 15 min at 4°C. The supernatant was collected as the Triton-soluble fraction, and a BCA protein assay was performed to determine protein concentration.

### Tau, NFL, and GFAP ELISA in plasma and brain lysates

Tau, NFL, and GFAP concentrations in plasma and brain lysates were measured in duplicate using commercially available enzyme-linked immunosorbent assay (ELISA) quantification kits from LS Bio (Seattle, WA) according to the manufacturer’s protocols (tau, LS-F23925; NFL, LS-F15886; GFAP, LS-F22386) by a technician who was unaware of the study group. In brief, plasma EDTA samples were diluted 2-fold in sample diluent prior to loading on the plate. Triton X-soluble brain lysates were first tested to ensure linearity and to determine the optimal loading concentration for each analyte. In the experimental cohort, 10ug TP was loaded per well to measure tau in Triton-soluble brain lysates, while 5ug protein was used for NFL, and 1ug TP was loaded per well to measure GFAP. Plates were read at 450 nm using a SpectraMax M5e multi-mode microplate reader.

### RNA preparation and qRT-PCR

A measure of 30–40 mg of frozen brain tissue was homogenized by hand in TRIzol, and total RNA was isolated using the RNeasy Plus Kit (Qiagen, Redwood City, CA) according to the manufacturer’s instructions with in-column DNase I treatment. RNA concentrations were measured on a NanoDrop, and 500 ng of RNA was transcribed to cDNA using the high-capacity cDNA reverse transcription kit according to manufacturer protocols (Applied Biosystems, Foster City, CA). cDNA was diluted 1:20 and added to a reaction mix (5 μL final volume) containing 100 nM gene-specific primers and SYBR GreenER qPCR SuperMix Universal (Thermo Fisher Scientific, Rockford, IL). All samples were run in triplicate and were analyzed on a QuantStudio™ 7 Flex Real-Time PCR System (Applied Biosystems, Foster City, CA) by a technician blinded to study group information. Relative quantification was determined using the ΔΔCt method and normalized to the endogenous control actin beta (*ACTB*). The following primers and their sequences were used: *ACTB* forward: 5’-CACGCCATCCTGCGTCTGGA-3′; *ACTB* reverse: 5’-AGCACCGTGTTGGCGTAGAG-3′; *MAPT*^3R^ forward: 5’-CGGGAAGGTGCAAATAGTCT-3′; *MAPT*^3R^ reverse: 5’-GTTATCCAGGGACCCGATCT-3′; *MAPT*^4R^ forward: 5’-GCGGCAGTGTGCAAATAGT-3′; *MAPT*^4R^ reverse: 5’-GGGACGTGGGTGATGTTATC-3′; *GFAP* forward: 5’-CTGGAGAGGAAGATCGAGTCTT-3′; *GFAP* reverse: 5’-ACGTCCATTTCCACGTGGACCT-3′; *IL6* forward: 5’-GATGCTTCCAATCTGGGTTCA-3′; *IL6* reverse: 5’-CATTTGTGGTGGGGTTAGGG-3′.

### Statistical analysis

The distribution of animal characteristics and operative variables was described by the median and the first (Q1) and third quartile (Q3) for metric variables. Due to the small sample size, it was not reasonable to assume that the data were normally distributed. Thus, the two-tailed Mann–Whitney U-test was used to compare the functional liver, kidney, and CBC parameters between the control group at baseline and the ALF group at the time points T_baseline_, T_24h_, and T_end_. Additionally, the Mann–Whitney *U*-test was used to evaluate the plasma concentration of GFAP, NFL, and tau proteins. The same test was used to compare the tissue protein concentrations and mRNA expressions of these markers in the FCTx and the Cb samples. Pearson’s correlations described the relationship between TP, AST, and ALT, and the plasma concentration of GFAP across all animals and time points. Statistical significance was defined by *p* ≤ 0.05.

## Result

The animal characteristics of all included animals are summarized in Table 1. The 85% liver resection was successfully performed in all ALF animals with minimal blood loss (230.0 mL). The mean survival time of the ALF animals was 43.0 h [40.75; 45.75]. Three of the four animals were euthanized after reaching the death-equivalent neurological endpoint, which was associated with elevated ICP above 20 mmHg for more than 1 h in association with rapid limb paddling, decerebrate posturing, dilated fixed pupils, and non-responsiveness to painful stimuli. These signs were consistent with cerebral edema and impending brain herniation in these animals. The remaining ALF animal survived to the 48 h endpoint.

**Table 1:** Animal characteristics and operative variables.

	ALF-group	Control-group
Animal number	4	6
Animal weight (kg)	29.8 [28.2; 33.27]	48.5 [47.1; 52.9]
Sex (female/male)	4/0	0/6
Pre-hepatectomy volume (mL)	966.0 [896.25; 1035.5]	-
Post-hepatectomy volume (mL)	101.9 [78.3; 125.75]	-
Percent volume resected (%)	89.2 [85.1; 92.8]	-
Resection blood loss (mL)	230.0 [177.5; 282.5]	-
Survival time (h)	43.0 [40.75; 45.75]	-
ICP-T_baseline_ (mmHg)	8.0 [7.0; 8.25]	-
ICP peak (mmHg)	17.5 [15.25; 20.0]	-

Values were described by their median and the first [Q1] and third [Q3] quartiles between the ALF group and the control group. ICP, intracranial pressure.

### Effect of 85% liver resection on the liver function parameter

The ALF induction by performing an 85% liver resection led to a significant increase in all metabolic, synthesis, and detoxification parameters, as shown in Table 2. The significant increase in AST and ALT values at the T_24h_ and T_end_ time points also indicated a remarkable liver injury following resection. Furthermore, the 85% liver resection was also associated with impaired liver synthesis and detoxification function, leading to an increase in serum ammonia concentration in the ALF animals. The declining synthesizing function of the liver also led to an increase in the INR values at T_24h_ and T_end_ time points. However, the ALF induction was not associated with a significant change in CBC parameters (*p* > 0.05) (Table 3).

**Table 2:** Functional liver and kidney parameters from both study groups (ALF and Control) at T_baseline_ and T_24_ and T_end_ point.

		ALF-group	*p*-value	Control-group
Alkaline phosphatase (U/L)	T_baseline_	131.5 [123.3; 146.3]	0.39	112.5 [96.7; 138.75]
	T_24h_	455 [416.0; 495.5]	**0.01**	
	T_end_	484.5 [458.7; 520.0]	**0.01**	
Alanine Transaminase (U/L)	T_baseline_	42.5 [38.25; 44.5]	0.67	41.0 [35.5; 42.0]
	T_24h_	73.0 [70.5; 78.3]	**0.01**	
	T_end_	63 [59.5; 73.7]	**0.01**	
Aspartate Transaminase (U/L)	T_baseline_	39.5 [35.0; 49.2]	0.20	30.0 [28.2; 34.7]
	T_24h_	873.5 [780.0; 1052.2]	**0.01**	
	T_end_	738.5 [590.7; 860.5]	**0.01**	
Total Bilirubin (mg/dL)	T_baseline_	0.35 [0.3; 0.4]	0.71	0.3 [0.3; 0.4]
	T_24h_	2.1 [1.8; 2.4]	**0.01**	
	T_end_	1.5 [1.1; 1.9]	**0.01**	
Creatinine (mg/dL)	T_baseline_	1.3 [1.05; 1.45]	0.91	1.3[1.1; 1.4]
	T_24h_	1.0 [0.9; 1.2]	0.83	
	T_end_	1.2 [1.1; 1.3]	0.23	
Total protein (g/dL)	T_baseline_	5.3 [5.12; 5.37]	0.02	6.3 [6.0; 6.5]
	T_24h_	4.5 [4.3; 4.7]	0.01	
	T_end_	4.4 [3.9; 4.7]	0.01	
Ammonia (μmol/L)	T_baseline_	63.0 [46.5; 79.5]	-	-
	T_24h_	185.0 [135.0; 191.5]	-	
	T_end_	233.0 [130; 353.25]	-	
INR	T_baseline_	1.1 [1.05; 1.12]	-	-
	T_24h_	1.6 [1.60; 1.62]	-	
	T_end_	1.6 [1.55; 1.72]	-	

Values were described by their median and the first [Q1] and third [Q3] quartiles and comparisons were made using the Mann–Whitney *U*-test. Bold indicated *p*-values smaller 0.05.

**Table 3:** Selected central blood count results from both study groups (ALF and Control) at T_baseline_, T_24h_, and T_end_ point.

		ALF-group	*p*-value	Control-group
Hemoglobin (g/dl)	T_baseline_	9.0 [8.1; 9.5]	0.73	8.5 [7.9; 9.0]
	T_24h_	8.7 [8.3; 8.9]	1.00	
	T_end_	7.9 [7.7; 8.6]	0.90	
Platelets (x10^9^/L)	T_baseline_	216 0.0 [190.0; 234.3]	0.90	223.0 [176.0; 294.0]
	T_24h_	227.5 [177.3; 280.3]	0.73	
	T_end_	154.5 [125.3; 217.0]	0.41	
Weight blood cells (x10^9^/L)	T_baseline_	14.1 [11.9; 17.0]	0.90	14.0 [13.4; 14.5]
	T_24h_	24.8 [21.2; 26.9]	0.29	
	T_end_	34.6 [24.0; 44.1]	**0.03**	
Neutrophils (x10^9^/L)	T_baseline_	6.3 [3.56; 8.2]	0.41	4.1 [3.9; 4.4]
	T_24h_	17.0 [12.6; 18.9]	0.03	
	T_end_	19.9 [9.1; 31.4]	0.03	

Values were described by their median and the first [Q1] and third [Q3] quartiles and comparisons were made using the Mann–Whitney *U*-test. Bold indicated *p*-values smaller 0.05.

### Evaluation of brain injury following 85% liver resection in plasma

**Figure 1:**
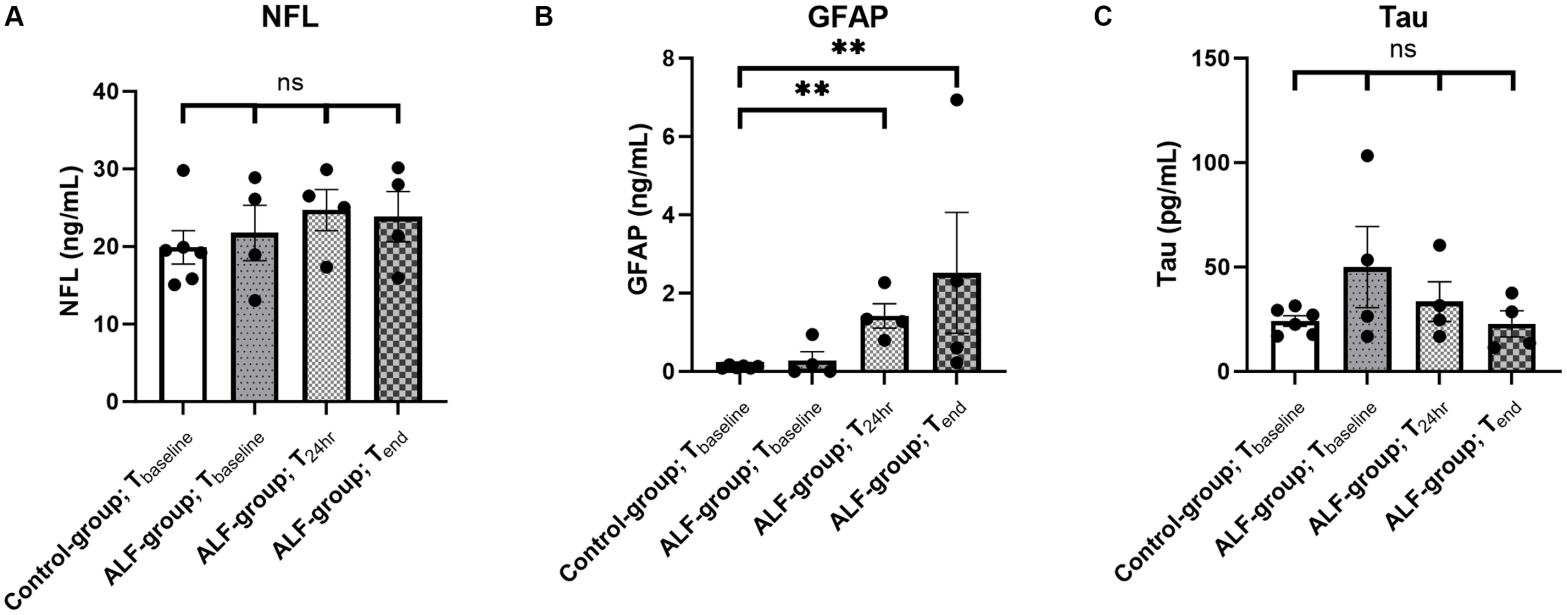
Plasma concentration of *NFL* **(A)**, *GFAP* **(B)**, and *tau* **(C)** in the ALF-group (*n* = 4) and control group animals (*n* = 6) at different time points. T_baseline_: baseline sample, T_24h_: sample taken 24 h following 85% liver resection; and T_end_: sample taken by reaching the predefined endpoint. ** *p*<0.01; ns: non significant.

**Figure 2:**
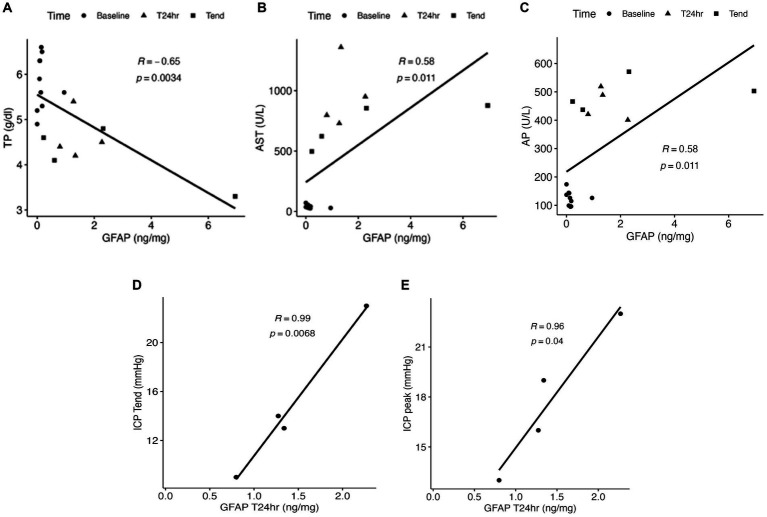
Correlation of total protein (TP) **(A)**, aspartate transaminase (AST) **(B)**, and alkaline phosphatase (AP) **(C)** to the plasma concentration of GFAP following the 85% liver resection. The correlation of ICP_end_ and ICP_peak_ measurements to the GFAP concentration at T_24h_ is indicated in **D** and **E**.

The ALF and control groups showed comparable concentrations of NFL, Tau, and GFAP prior to the 85% liver resection (T_baseline_: p_NFL_ = 1; p_Tau_ = 0.61; p_GFAP_ = 1; Figure 1). In contrast, a significant elevation in the plasma concentration of GFAP was observed in the ALF group at the T_24h_ and T_end_ time points compared to the control group at T_baseline_ (Figure 1B). The median GFAP concentration in the ALF group was 1.31 ng/mL, compared to 0.11 ng/mL in the control group (*p* < 0.05). At the time the animals reached the endpoint, the ALF group showed an increase in GFAP to 1.46 ng/mL (*p* < 0.05). Notably, we also observed a significant correlation between plasma GFAP levels and TP in serum (Pearson *r* = −0.65, *p* = 0.003; Figure 2A), as well as serum concentrations of AST (Pearson *r* = 0.58, *p* = 0.01; Figure 2B), AP (Pearson *r* = 0.58, *p* = 0.01; Figure 2C), ICP_peak_ (Pearson *r* = 0.99, *p* = 0.0068; Figure 2D), and ICP_end_ (Pearson *r* = 0.96, *p* = 0.04; Figure 2E). These findings indicate that peripheral GFAP levels are associated with ALF-induced brain injury in a porcine model.

### Evaluation of the anterior pole of the frontal cortex (FCTx) and the cerebellum (Cb) by using ELISA and qRT-PCR techniques

**Figure 3:**
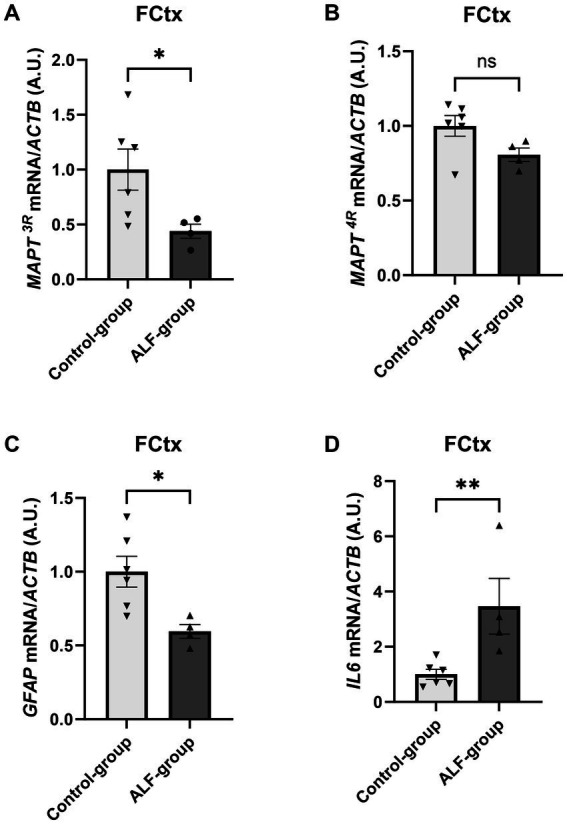
qRT-PCR results of the frontal cortex (FCTx) comparing the RNA expression of *MAPT*^3R^ **(A)**, *MAPT*^4R^ **(B)**, *GFAP* **(C)**, and *IL6* **(D)** normalized to the endogenous control (*ACTB:* actin beta) between the ALF group (*n* = 4) and the control group (*n* = 6). *, *p* < 0.05; **, *p* < 0.01. ns: non significant.

To assess molecular changes in the brain of this porcine ALF model, we first examined NFL, GFAP, and tau protein expression by ELISA. However, we did not observe any significant differences between the control and ALF groups in either the FCTx or the Cb (Supplementary Figure S1). We then used qRT-PCR to examine RNA expression. Given that alternative splicing of the tau gene (MAPT) can result in various isoforms, with an imbalance between three-repeat (3R) and four-repeat (4R) containing isoforms linked to neurodegeneration (24– 26), we generated primers to detect porcine *MAPT*^3R^ and *MAPT*^4R^ RNA transcripts. Of interest, *MAPT*^3R^ expression was significantly decreased in the FCTx of ALF animals (*p* < 0.05, Figure 3A), and *MAPT*^4R^ levels were trending toward a reduction (*p* = 0.07, Figure 3B). In contrast, no statistically significant differences in *MAPT*^3R^ or *MAPT*^4R^ mRNA levels were detected in the Cb (Supplementary Figures S2A,B).

Given the observed relationship between plasma GFAP levels and ALF-associated brain injury, we also wanted to examine *GFAP* mRNA expression in the brain. Consistent with reports in human ALF patients (24) and a rat model of ALF (25), we found a significant reduction in *GFAP* levels in the FCTx in ALF animals compared to controls (*p* = 0.018, Figure 3C). Intriguingly, there was no significant change in *GFAP* expression in the Cb of ALF animals (Supplementary Figure S2C), which might suggest this brain region is less susceptible to ALF-induced injury. Finally, as neuroinflammation is a well-known feature of ALF (26), we measured *IL6* mRNA expression. This analysis indicated a strong 2.8-fold upregulation of *IL6* expression in the FCTx (*p* = 0.018, Figure 3D) but not in the Cb of ALF animals (Supplementary Figure S2D), supporting the idea that the FCTx is more sensitive to ALF-induced brain injury than the CB.

## Discussion

The current study evaluated blood concentrations of GFAP, NFL, and tau proteins in an established preclinical ALF model (23). From induction to ALF-induced brain herniation, the different concentrations of these proteins were evaluated and compared to healthy controls. In this comparison, plasma GFAP levels increased significantly in the ALF model 24 h after induction and continued to increase until reaching the study’s endpoint, indicating that blood GFAP can be used as a biomarker for brain injury associated with ALF. The subsequent statistical analysis also identified a strong correlation of plasma GFAP level with parameters of liver injury (AST and ALT) and synthesis (TP), further supporting the use of blood GFAP as a useful tool for the early detection of ALF-associated brain damage. While previous studies have shown that elevated blood GFAP concentration is a suitable parameter for predicting unfavorable outcomes in patients with moderate to severe traumatic brain injury (27), the use of GFAP in the early detection and diagnosis of ALF-associated brain injury in humans or large animals has not been well explored. A recent study showed elevated GFAP in the blood of patients with hepatic encephalopathy associated with cirrhosis, which was correlated with ammonia and IL6 levels in serum (21).

The current porcine model will allow us to confirm and consolidate previous findings while also providing new insights from the current study. Notably, Chen et al. demonstrated that elevated serum IL6 levels are observed in the development of ALF using a similar porcine 85% liver resection model (23). As such, it will be interesting in future studies to investigate the relationship between GFAP and IL6 levels in the blood and to further assess whether the combination of these biomarkers provides greater diagnostic and prognostic insight.

Currently, the correlation between GFAP concentration in brain tissue and the bloodstream is complex and not well understood. Especially, the release of GFAP from the cells into the bloodstream under physiological and pathological conditions is complex and still under debate (10). For the GFAP protein concentration in brain tissue, the present study demonstrated that GFAP in both the FCTx and the CB is comparable between the ALF and healthy control groups. However, we also found that *GFAP* RNA expression was significantly reduced in the FCTx of ALF animals compared to the healthy controls, which is consistent with reports in human ALF patients (24) and a rat model of ALF (25). As *GFAP* RNA was unchanged in the Cb, our findings may indicate that the FCTx is more susceptible to ALF-induced brain injury than the Cb. This represents a notable observation since human studies have not mentioned the different susceptibility between the FCTx and the CB patients with hepatic encephalopathy. However, human studies have only examined patients with end-stage cirrhosis as a cause of hepatic encephalopathy, which may have a potentially different impact on FCTx and CB due to the chronic nature of the disease (28–30). Therefore, further studies need to verify these results in more detail, including an analysis of the exact mechanistic background of GFAP RNA expression in relation to the different anatomical brain regions. If there is an additional release of GFAP from glial cells in the peripheral or enteric nervous system is also a matter for discussion. In particular, GFAP release from enteric glial cells could be triggered by the change in splanchnic circulation associated with extended liver resection (31). However, further studies are needed to investigate the release of GFAP from enteric glial cells in the context of extended liver resection and the changes in the splanchnic circulation. In contrast to the tissue analysis, the bloodstream concentration of GFAP correlates strongly with TP, AST, ALT, total bilirubin, and AP levels after 24 h of ALF, which is also of high scientific and clinical value. Additionally, the prediction value of GFAP at the T_24h_ time point also correlates with both the ICP_peak_ and ICP_end_ values. First, the high levels of these proteins and enzymes in combination with the elevated INR values confirm the successful induction of ALF in this model (32). Second, the results illustrate the close link between the cellular mechanisms of the liver and brain. GFAP may play an important role in activating hepatic stellate cells, leading to vascular remodeling in the liver following acute injury (31), which in the current context occurs after acute damage. However, in the central nervous system, the mechanism is different, where GFAP expression by astrocytes is required for blood–brain barrier repair after injury (33, 34). The associated diagnostic significance of this protein in the context of acute liver and brain damage is thus evident.

While we did not observe any changes in blood concentrations of tau in ALF animals in the current study, we found that changes in *MAPT*^3R^ and *MAPT*^4R^ RNA expression are associated with ALF-induced brain injury in the FCTx is intriguing. In particular, tau aggregation is a neuropathological hallmark of several neurodegenerative disorders classified as tauopathies, including frontotemporal dementia and Alzheimer’s disease, and changes in splicing of the *tau* gene leading to an imbalance in *MAPT*^3R^ and *MAPT*^4R^ isoforms have been implicated in neurodegeneration (35–37). As such, future studies will need to assess changes in *tau* splicing in different regions of the brain, as well as examine whether changes in tau solubility or phosphorylation status are observed. Given that the amygdala and hippocampus are highly susceptible to degeneration with aging, it would be particularly intriguing to investigate potential alterations in tau in these brain regions following ALF.

The current study searching for future biomarkers for identifying brain injury showed promising results. Especially GFAP in plasma and brain tissue was found to be a new reliable marker for ALF-associated brain injury. However, study-specific limitations need to be considered in interpreting the results. Due to the small sample size of animals, which leads to high variation in the investigated parameters, the age-dependent size difference may potentially affect the concentration of specific proteins, especially in the brain tissue itself. Furthermore, potential gender-specific effects in the respective groups cannot be ruled out when interpreting the results. Therefore, future studies may need larger group sizes to reduce the variation of the results and confirm study readouts.

Furthermore, the use of an 85% liver resection model in these large animals represents only one approach to inducing ALF. Confirmation of these results in alternative drug- or ischemia-induced ALF models (38) as well as in age- and sex-randomized studies is essential for establishing GFAP as a new innovative biomarker for liver injury-related brain parameters. Additionally, placing the ICP probe in the brain parenchymal area could also potentially affect the concentration of selective proteins. While available data have not identified such a correlation, a potential effect cannot be conclusively ruled out. Moreover, ICP monitoring remains the gold standard for assessing ICP and, in our opinion, is required for the objective comparison of ICP in preclinical ALF models.

Despite these limitations, the data presented are promising and, for the first time, demonstrate the correlation of brain injury parameters in a large ALF animal model, warranting further studies to investigate the mechanisms of the liver–brain axis.

## Conclusion

The current report demonstrates that GFAP represents a promising new biomarker in the context of liver-associated brain injury. In addition to already established markers such as S100B, GFAP can be an additional tool for the prediction and monitoring of brain injury in patients with ALF. Further research should focus on these interesting observations and advance the development of a new brain injury score integrating various non-invasive parameters such as serum biomarkers or ONSD. This could avoid the invasive measurement of intracranial brain pressure in the future, especially for patients with severe comorbidities.

## Data availability statement

The original contributions presented in the study are included in the article/Supplementary material, further inquiries can be directed to the corresponding authors.

## Ethics statement

The animal study was approved by Institutional Animal Care and Use Committee. The study was conducted in accordance with the local legislation and institutional requirements.

## Author contributions

PF: Conceptualization, Formal analysis, Investigation, Project administration, Visualization, Writing – original draft. SH: Writing – review & editing. LF: Formal analysis, Methodology, Writing – review & editing. BPA: Methodology, Project administration, Supervision, Writing – review & editing. AM: Data curation, Investigation, Project administration, Writing – review & editing. BA: Investigation, Writing – review & editing. ZQ: Software, Writing – review & editing. SW: Writing – review & editing. IA: Formal analysis, Writing – review & editing. SN: Conceptualization, Data curation, Funding acquisition, Project administration, Resources, Supervision, Visualization, Writing – review & editing. CC: Conceptualization, Formal analysis, Funding acquisition, Investigation, Project administration, Resources, Supervision, Validation, Visualization, Writing – original draft, Writing – review & editing.
