# Supplementary material for: Comprehensive analysis of brain injury parameters in a preclinical porcine model of acute liver failure
Source: Front Med (Lausanne). 2024 Mar 28;11:1363979. doi: 10.3389/fmed.2024.1363979 (PMC11007081; doi:10.3389/fmed.2024.1363979)
Supplement: Supplementary file 1 [file Data_Sheet_1.PDF]

**Supplementary material**

**Comprehensive analysis of brain injury parameters in a preclinical porcine model of acute liver failure**

Philipp Felgendreff[1,2], Seyed M. Hosseiniasl[1], Lisa Felgendreff[3], Bruce P. Amiot[1], Anna Minshew[1], Boyukkhanim Ahmadzada[1], Zhi Qu[4], Silvana Wilken[1], Ines Arribas Gomez[5], Scott L. Nyberg[1,6†*], Casey N. Cook[6†*]

[†] These authors have contributed equally to this work and share senior authorship

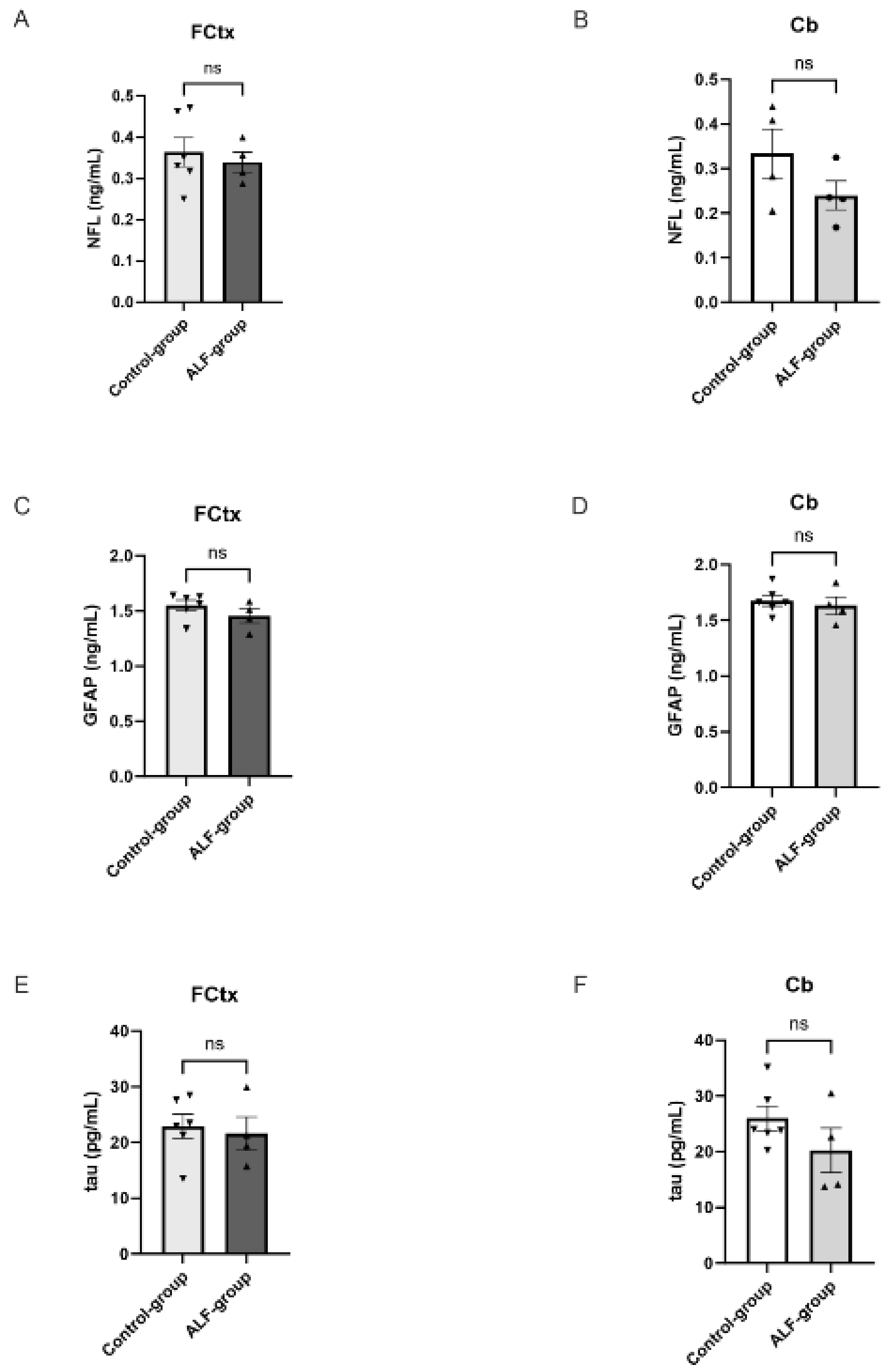


Supplementary Figure 1: Protein concentration of *NFL* in the frontal lobe (FCtx) (A) and cerebellum tissue (Cb) (B), *GFAP* in FCtx (C) and Cb (D) and *tau* in FCtx (E) and Cb (F), ns: non-significance, Proteins were detected using ELISA techniques.

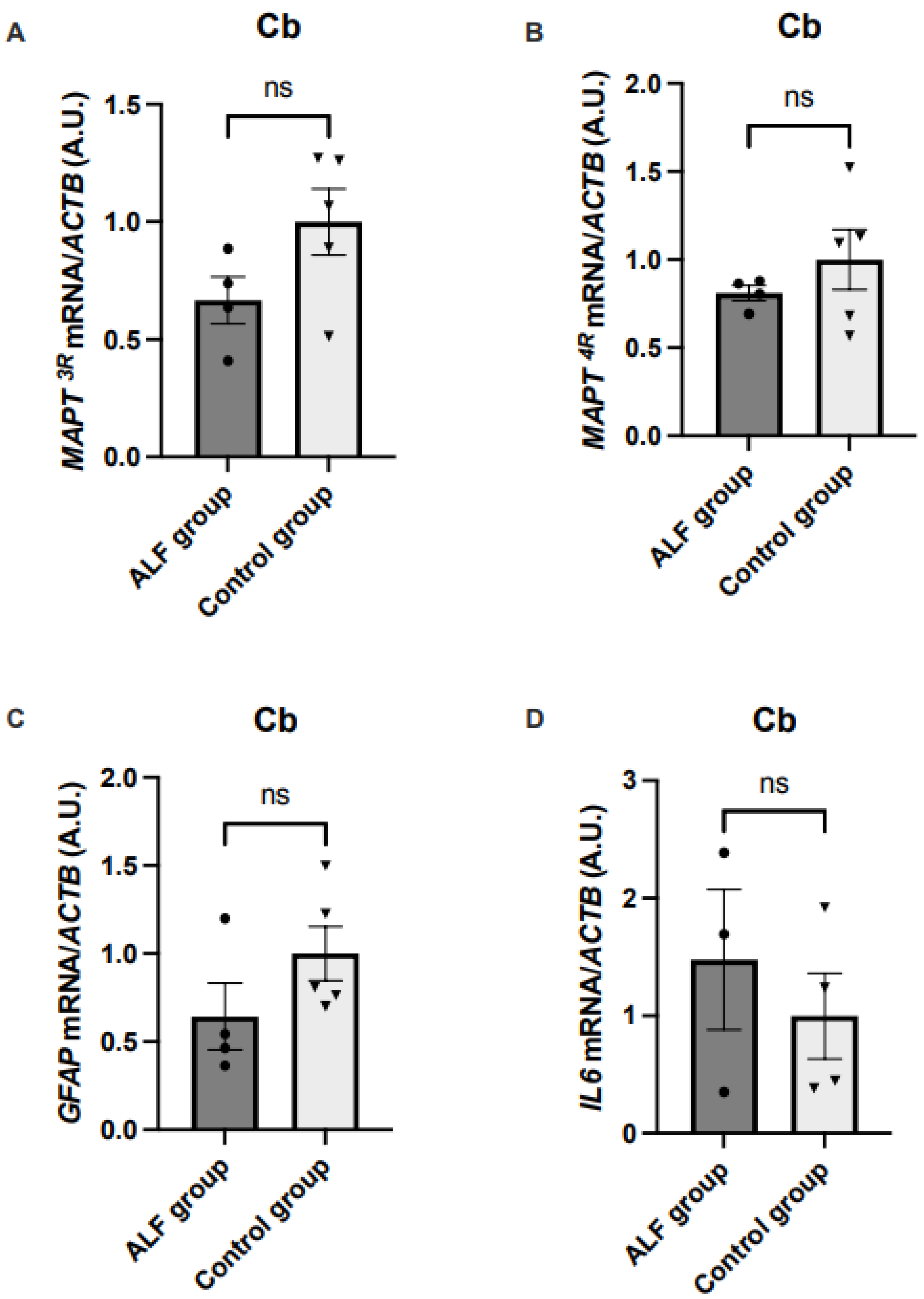


Supplementary Figure 2: qRT-PCR results of the cerebellum (Cb) comparing the RNA expression of *MAPT*$^{3R}$ (A), *MAPT*$^{4R}$ (B), *GFAP* (C), and *IL6* (D) normalized to the endogenous control (*ACTB*) between the ALF-group and the control-group. ns: non-significant
